# Preoperative low absolute lymphocyte count to fibrinogen ratio correlated with poor survival in nonmetastatic colorectal cancer

**DOI:** 10.1186/s12957-022-02775-z

**Published:** 2022-09-24

**Authors:** Xiang Huang, Yu Huan, Long Liu, Qianwen Ye, Jian Guo, Bing Yan

**Affiliations:** 1 Department of Pulmonary Function Test, Shanghai Pulmonary Hospital, Tongji University School of Medicine, Shanghai, 200443 People’s Republic of China; 2 Department of Clinic Laboratory, Hainan Hospital of Chinese PLA General Hospital, Sanya, Hainan Province 572000 People’s Republic of China; 3 Department Traditional Chinese Medicine, Tianyou Hospital of Tongji University, Shanghai, 200331 People’s Republic of China; 4 Department of Oncology, Hainan Hospital of Chinese PLA General Hospital, Sanya, Hainan Province 572000 People’s Republic of China

**Keywords:** Colorectal cancer, Lymphocyte count, Fibrinogen, Disease-free survival, Overall survival

## Abstract

**Background:**

Preoperative absolute lymphocyte count (LC) and fibrinogen (FIB) are useful prognostic indicators in colorectal cancer (CRC). However, the prognostic value of the LC to FIB ratio (LFR) has never been addressed.

**Methods:**

A total of 189 nonmetastatic CRC patients after resection were enrolled retrospectively. The significance of the LFR in predicting disease-free survival (DFS) and overall survival (OS) was estimated by receiver operating characteristic curve analysis, and the prognostic efficacy was compared with individual LC and FIB. Patients were assigned to LFR low or high subgroups. Differences in clinicopathological features among these subgroups were calculated, and the survival differences of these subgroups were determined by the Kaplan-Meier analysis. A Cox proportional hazards model was applied to test the risk factors for survival.

**Results:**

Taking 0.54 as the optimal cutoff point, the LFR had sensitivities of 79.70% and 86.40% and specificities of 52.30% and 51.00% in predicting the DFS and OS, respectively. A total of 109/189 (57.67%) patients were assigned to the LFR low group, and these patients were more likely to be characterized by criteria such as T_3_ + T_4_ (*P* < 0.01), stage 3 (*P* < 0.01), tumor deposits (*P* = 0.01), high CEA (*P* < 0.01), or CA19-9 levels (*P* = 0.04). And they also displayed worse DFS (log rank = 18.57, *P* < 0.01) and OS (log rank = 20.40, *P* < 0.01) than the high LFR group. Finally, the LFR was independently associated with inferior DFS (*HR* = 0.32, 95% *CI*: 0.16–0.61, *P* < 0.01) and OS (*HR* = 0.23, 95% *CI*: 0.09–0.55, *P* < 0.01).

**Conclusions:**

The LFR is a useful prognostic indicator in nonmetastatic CRC, and patients with a relatively low LFR had poor survival.

Colorectal cancer (CRC) is still a major cause of cancer-related death worldwide [1]. In contrast to the USA, in which the age-standardized incidence and mortality rates of the disease have decreased noticeably in recent years, the incidence rate is still increasing in China [2]. Although the majority of early stage cases can be cured by surgery or surgery plus adjuvant chemotherapy (AC) [3], over a third of patients will die within 5 years [4]. Developing for a reliable and easily accessible prognostic indicator is still important in practice, particularly for the determination of therapeutic strategies.

Cancer-associated inflammation is regarded as one of the hallmarks of cancer [5] and plays an essential role at different stages of cancer development [6]. The elevated cytokines and chemokines in the inflammatory environment can alter not only the proportions of inflammatory cells [7, 8] but also their functions [9]. Lymphocytes are an important component of leukocytes and are the main player in adaptive anticancer immunity [10]. Lymphocytes have profound effects in many aspects of cancer, such as inhibiting their occurrence [11], preventing dissemination [12] or recurrence [13], and regulating treatment response [14]. Not unexpectedly, the count of these cells in peripheral blood as well as in the tumor microenvironment (TME) was also found to have an important role in prognosis in many malignancies [15–17] including CRC [18–20]. Taking into consideration that the altered proportion of leukocytes in the inflammatory environment would also be meaningful in reflecting the anticancer immune response, a series of new prognostic indicators were established to further improve the prognostic efficacy based on absolute lymphocyte count (LC) in CRC, including the neutrophil to lymphocyte ratio (NLR: defined as the absolute number of neutrophils divided by the number of lymphocytes) [21], lymphocyte to monocyte ratio (LMR: defined as the absolute number of lymphocytes divided by the number of monocytes) [22], and LANR (defined as the absolute number of lymphocytes multiplied by the level of albumin and divided by the absolute number of neutrophils) [23].

Interestingly, some inflammation-related proteins were also found to be prognostically meaningful in addition to these inflammatory cells. Fibrinogen (FIB), which is a glycoprotein that is mainly synthesized by the liver as an acute-phase response, was previously thought to play a role mainly in coagulation [24]. However, it was found that FIB could also be released by cancer cells [25] and involved in many other biological processes including tumor angiogenesis, cancer cell proliferation, adhesion, and migration [26, 27]. Based on these data, mounting evidence indicates that a frequently elevated FIB in cancer patients is associated with poor survival [28–33] which includes CRC [34, 35]. Nonetheless, it is worth noting that neither single LC nor single FIB was sufficient to provide a precise prediction of the prognosis in CRC. As previous studies have indicated, the area under the curves (AUCs) for individual LC in predicting the outcome ranged from 0.58 to 0.61 with a relatively low sensitivity or specificity [19, 36]. In line with this, the AUC for FIB in predicting overall survival (OS) was only 0.57 [37], and the optimal cutoff points were highly inconsistent in these studies for both LC and FIB [19, 34, 38]. Therefore, it is plausible that a combination of these two indicators, namely, the LC to FIB ratio (LFR) could be more reliable in prognosis for CRC patients. However, there is currently little research on the LFR in CRC.

In this study, we aimed to explore the prognostic value of LFR and compare its prognostic efficacy with individual LC and FIB. Further, we tested the usefulness of LFR in normal carcinoembryonic antigen (CEA) cases in CRC.

## Methods

### Study population

Data from patients who received radical resection of the primary lesion at the Hainan Hospital of Chinese PLA General Hospital were retrospectively collected from December 2012 to June 2020. Those who met any one of the following criteria were excluded: (1) any preoperative neoadjuvant therapies, (2) evidence of distant metastasis by imaging examinations, (3) in situ lesions or active immune system diseases, (4) the usage of any anticoagulant drugs, (5) lacking preoperative laboratory results for blood or coagulation function tests, (6) lacking any of pathological TNM information, and (7) lacking reliable follow-up or a follow-up duration less than 3 years (y)/36 months (m). Other data, including tobacco or alcohol use history and complications (mainly hypertension and type 2 diabetes), were collected as described previously [39–41]. Tumor stage was followed by the seventh edition of the American Joint Committee on Cancer staging manual. The study was performed in line with the principles stated in the Declaration of Helsinki and was approved by the ethics committee of the Hainan Hospital of Chinese PLA General Hospital (ID: 301HLFYLS15). Patients or their relatives authorized provided the informed consent.

### Definition of LFR and other prognostic indicators

Peripheral venous blood was collected between 6:00 am and 9:00 am before breakfast within 1 week before the operation and processed in clinical laboratory center as described previously [40]. The blood sample was centrifuged at 3000–3600 r/min (ST-16, Thermo Fisher Scientific, USA), and upper plasma was then tested for tumor markers (CEA: 0–5 μg/ml, CA19-9: 0–37 μg/ml) using the electrochemiluminescence method with the automatic analysis system (Cobas e 601, Roche, Switzerland) and FIB using the Fibrinogen-C XL Kit according to the manufacturer’s instructions (ACL TOP 700, A Werfen Company, USA). The specific cell fraction in blood was analyzed using an automatic blood cell analyzer (XN3000, Sysmex Corporation, Japan). The blood sample was placed in the analyzer where a portion of it was automatically diluted to a 1:60 dilution and lysed by the addition of the special Sysmex lysing reagent (Lysercell WDF). Fluorocell WDF was then added, and the entire dilution was maintained at a constant temperature for a defined time period to label the nucleated cells in the sample. The labeled sample was then moved into the sheath flow detector where side scattered light and side fluorescence were measured allowing the LC to be computed. The LFR was calculated by the absolute number of LCs divided by the level of the FIB and then divided by 10^9^ to facilitate the data input. Other established prognostic indices, including NLR, LMR, platelet counts to lymphocyte ratio (PLR: defined as the absolute number of lymphocytes divided by the number of platelets), and prognostic nutritional index (PNI: defined as the level of albumin plus 5 multiplied by the absolute number of lymphocytes and then divided by 10^9^), were also collected as previously described [21, 22, 42].

### Definition of disease-free survival (DFS) and OS

The follow-up was conducted as described previously [40] and routine laboratory tests and imaging examinations including computed tomography, magnetic resonance imaging, and ultrasonography were performed in this period. DFS was defined from the date of surgery to the date of any recurrence or metastasis or the date of death from any cause, and OS was defined from the same point to the point of any cause of death. The latest follow-up point ended in December 2021.

### Statistical analysis

All statistical analyses were performed using SPSS 20.0 (SPSS Inc., Chicago, IL, USA), MedCalc v19.0.7 (MedCalc Software Ltd., Ostend, Belgium), and GraphPad Prism 5 (GraphPad Software Inc., San Diego, CA, USA). Receiver operating characteristic curve (ROC) analysis was used to identify the predicting efficacy of LFR for DFS and OS, with an optimal discriminator point to check the sensitivity and specificity. In addition, the AUC of LFR was compared with individual LC and FIB. The relationship of the LFR with the NLR, LMR, PLR, and PNI was determined by the Pearson correlation coefficient. Patients were assigned to LFR low or high subgroups based on the optimal discriminator point, and the differences in clinicopathological data among these subgroups were using a *χ*^2^ test or Student’s *t*-test. DFS and OS differences between LFR low and high subgroups were estimated by a Kaplan-Meier analysis followed by log-rank tests. Risk factors for survival were checked using the Cox proportional hazards model. All tests were two sided with *P* < 0.05 regarded as statistically significant, and all the results were kept to a maximum of two decimal places.

## Results

### Patients’ demographics and the prognostic efficacy of LFR

**Fig. 1:**
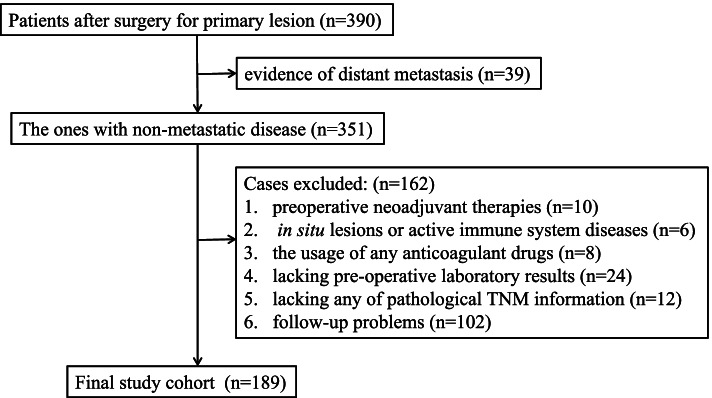
Flow chart of the study

**Fig. 2:**
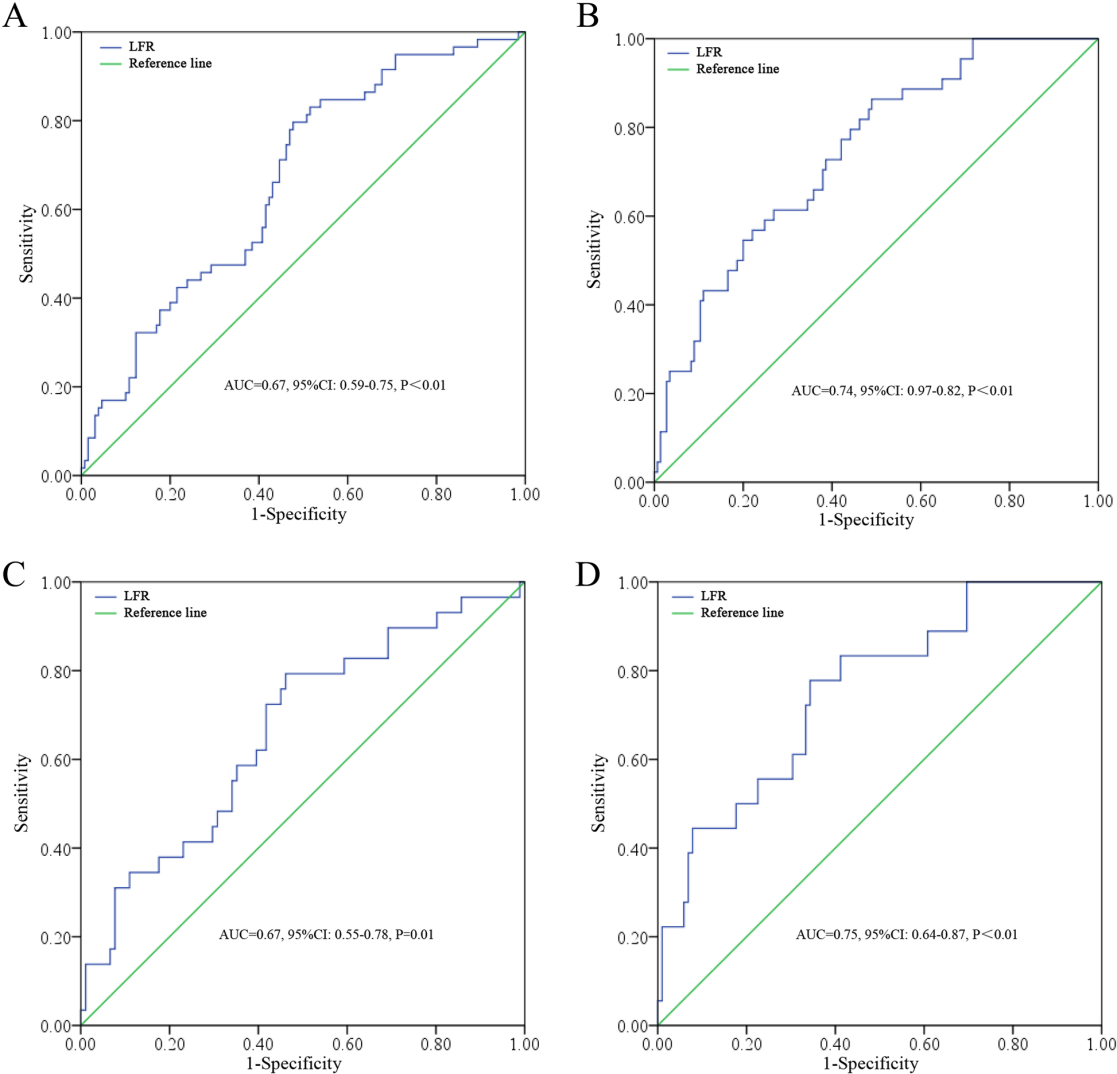
The ROC analysis results of LFR in predicting DFS (**A**), OS (**B**) in the study cohort and in predicting DFS (**C**), and OS (**D**) in CEA normal patients

A total of 189 patients were included in the study (Fig. 1) with 40, 77, and 72 stages 1, 2, and 3 cases, respectively. During the follow-up, 2 patients in stage 1, 17 patients in stage 2, and 25 patients in stage 3 died, and the 3-year (y) overall survival rate was 76.72% (145/189). The mean age of these patients was 59.61 years (range: 26–85 years). And the mean follow-up period was 64.40 m (range: 1–114 m). By ROC tests, the LFR had sensitivities of 79.70% and 86.40% and specificities of 52.30% and 51.00% in predicting the DFS (*AUC* = 0.67, *P* < 0.01) and OS (*AUC* = 0.74, *P* < 0.01), respectively (Fig. 2 A–B). The prognostic efficacy of LFR was superior to LC (*AUC* = 0.68, *Z* = 2.04, *P* = 0.04) or FIB (*AUC* = 0.66, *Z* = 2.14, *P* = 0.03) alone for OS but not for DFS (LC: *AUC* = 0.63, *Z* = 1.04, *P* = 0.30; FIC: *AUC* = 0.58, *Z* = 1.95, *P* = 0.05). Interestingly, the prognostic efficacy of LFR was also significant in CEA normal cases both for DFS (*AUC* = 0.67, *P* = 0.01) and OS (*AUC* = 0.75, *P* < 0.01; Fig. 2 C–D).

### Correlation of LFR with NLR, LMR, PLR, and PNI

**Fig. 3:**
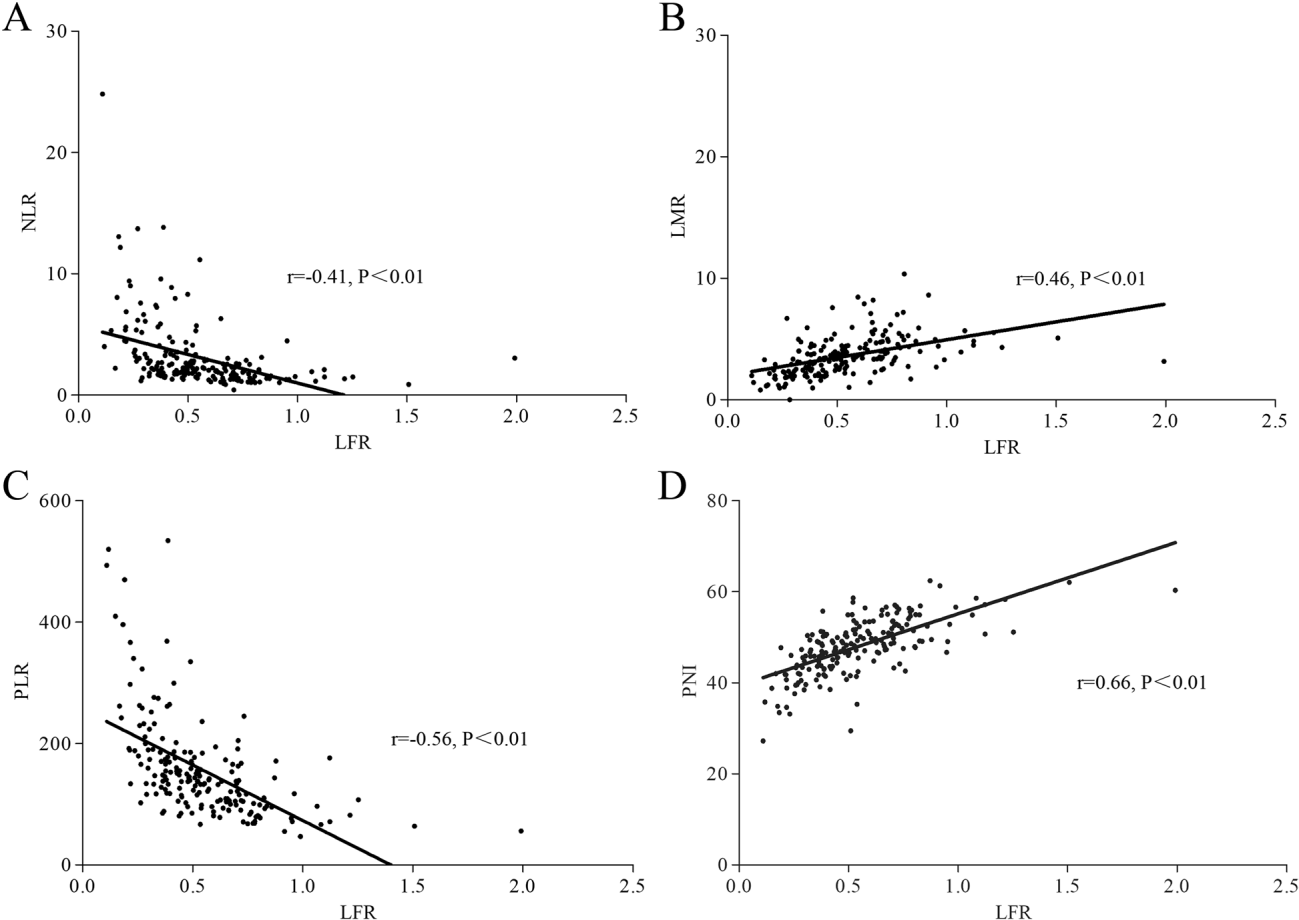
Correlation of LFR with other prognostic indices, such as NLR (**A**), LMR (**B**), PLR (**C**), and PNI (**D**)

Using a Pearson correlation analysis, we found a significant positive correlation between LFR and LMR (*r* = 0.46, *P* < 0.01) and LFR and PNI (*r* = 0.66, *P* < 0.01) and a negative correlation between LFR and NLR (*r* = −0.41, *P* < 0.01) and LFR and PLR (*r* = −0.56, *P* < 0.01). The strengths of these correlations were moderate, with the LFR and PNI being the strongest (Fig. 3).

### Differences in clinicopathological parameters among LFR subgroups

Taking 0.54 as the optimal cutoff point according to the *Youden index* in ROC tests, patients were assigned to the LFR low (< 0.54) or high (≥ 0.54) subgroups, and patients in the LFR low group were more likely to be characterized by criteria such as T_3_ + T_4_ (*P* < 0.01), stage 3 (*P* < 0.01), tumor deposits (*P* = 0.01), high CEA (*P* < 0.01), or CA19-9 levels (*P* = 0.04; Table 1).

**Table 1:** Differences in clinicopathological parameters among LFR low or high subgroups

	Patient no.	LFR		
		Low	High	*P*
**Age (y)**				0.11
< 60	88	45	43	
≥ 60	101	64	37	
**Gender**				0.54
Male	119	71	48	
Female	70	38	32	
**Tobacco use history**				0.20
Never	132	72	60	
Current + former	57	37	20	
**Alcohol use history**				0.13
Never	117	62	55	
Current + former	72	47	25	
**Hypertension**				0.86
Without	150	86	64	
With	39	23	16	
**Type 2 diabetes**				0.63
Without	169	96	73	
With	20	13	7	
**Tumor sites**				0.62
Right	52	28	24	
Left	137	81	56	
**Histological grade**				0.22
Well + moderate	160	89	71	
Poor	29	20	9	
**Mucinous constituent**				0.25
Without	156	93	63	
With	33	16	17	
**Tumor deposits**				0.01*
Without	172	94	78	
With	17	15	2	
**CEA level**				< 0.01*
Normal	120	60	60	
Elevated	69	49	20	
**CA19-9 level**				0.04*
Normal	160	87	73	
Elevated	29	22	7	
**Combined T stages**				< 0.01*
T_1_ + T_2_	52	16	36	
T_3_ + T_4_	137	93	44	
**Combined N stages**				0.13
N_0_	115	61	54	
N_1 +_ N_2_	74	48	26	
**TNM stages**				< 0.01*
I	40	12	28	
II	77	50	27	
III	72	47	25	
**BMI (kg/m**^**2**^**)**	189	22.95 ± 3.62	23.83 ± 3.42	0.09

*With significant statistical difference

### Survival differences in LFR subgroups

**Fig. 4:**
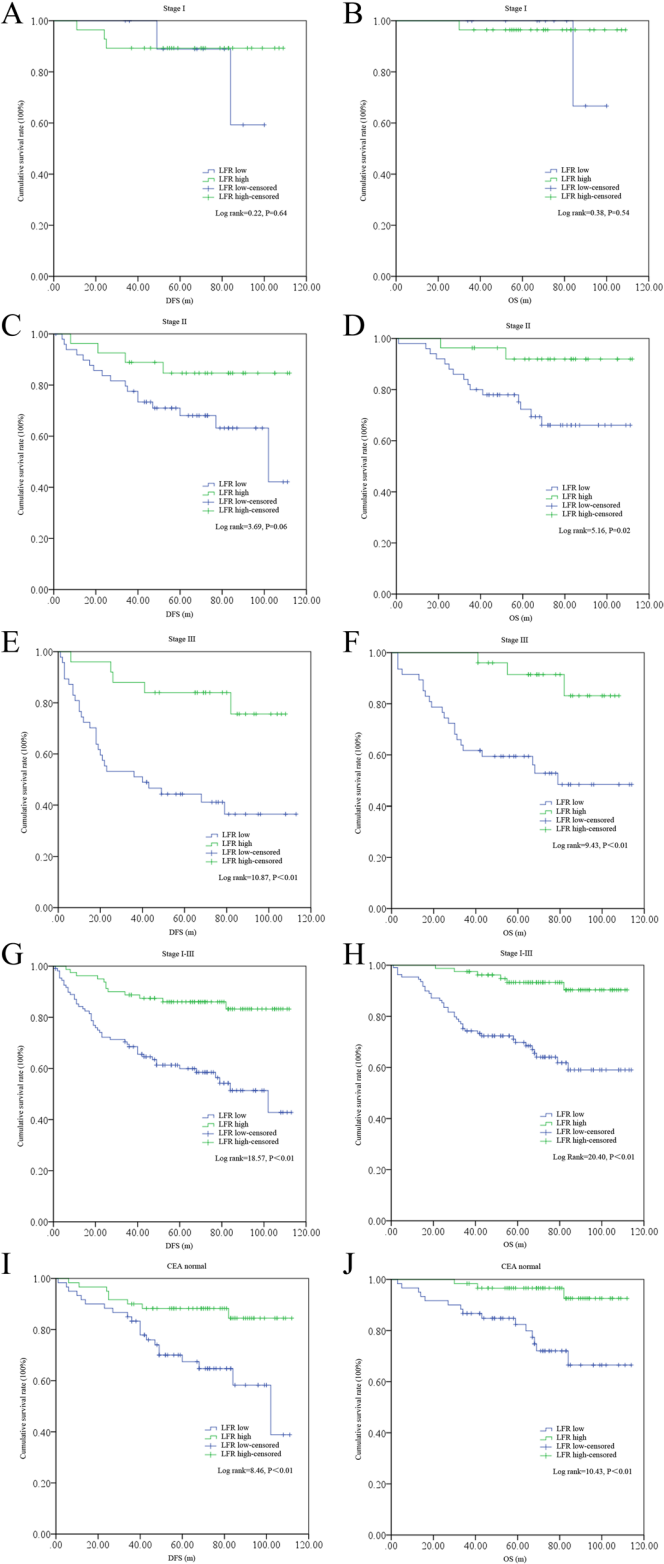
The survival differences between the LFR low and high subgroups. DFS differences in stages 1 (**A**), 2 (**C**), 3 (**E**), the whole cohort (**G**), and CEA normal patients (**I**); OS differences in stages 1 (**B**), 2 (**D**), 3 (**F**), the whole cohort (**H**), and CEA normal patients (**J**)

Using a Kaplan-Meier analysis, we found that patients in the low LFR group displayed an obviously worse DFS in stage 3 and OS in stages 2–3 than those in the high LFR group (Fig. 4 A–F). Patients with a relatively low LFR showed significantly poorer DFS (log rank = 18.57, *P* < 0.01) and OS (log rank = 20.40, *P* < 0.01) than those with a high LFR in the whole cohort (Fig. 4 G–H). Additionally, the DFS (log rank = 8.46, *P* < 0.01) and OS (log rank = 10.43, *P* < 0.01) were also worse in those patients with a normal CEA level (Fig. 4 I–J).

### Univariate and multivariate tests of risk factors for DFS and OS

Using univariate analysis, gender, tumor deposits, CEA, or CA19-9 levels, combined T and N stages, TNM stages, BMI, and LFR, were found to be significant risk factors for both DFS and OS; additionally, age and histological grade were found to also be significant risk factors for OS (Table 2). When these factors (only *P* < 0.05) were integrated into multivariate analysis, the LFR was found to be an independent risk factor for both DFS (*HR* = 0.32, 95% *CI*: 0.16–0.61, *P* < 0.01) and OS (*HR* = 0.23, 95% *CI*: 0.09–0.55, *P* < 0.01) (Table 3).

**Table 2:** Risk factors for DFS and OS by univariate analysis

	DFS			OS		
	***P***	HR	95% ***CI***	***P***	HR	95% ***CI***
**Age (years)**						
< 60	1			1		
≥ 60	0.56	1.17	0.69–1.95	0.02*	2.17	1.14–4.16
**Gender**						
Male	1			1		
Female	0.03*	0.53	0.30–0.95	0.03*	0.46	0.23–0.93
**Tobacco use history**						
Never	1			1		
Current + former	0.93	1.02	0.59–1.77	0.80	1.09	0.57–2.08
**Alcohol use history**						
Never	1			1		
Current + former	0.11	1.52	0.91–2.53	0.25	1.42	0.78–2.57
**Hypertension**						
Without	1			1		
With	0.94	1.03	0.54–1.93	0.42	1.33	0.67–2.62
**Type 2 diabetes**						
Without	1			1		
With	0.34	1.65	0.60–4.55	0.78	1.16	0.41–3.23
**Tumor sites**						
Right	1			1		
Left	0.36	0.75	0.41–1.39	0.34	0.70	0.34–1.45
**Histological grade**						
Well + moderate	1			1		
Poor	0.13	1.63	0.87–3.08	0.03*	2.12	1.07–4.20
**Mucinous constituent**						
Without	1			1		
With	0.60	1.19	0.62–2.30	0.41	1.37	0.66–2.84
**Tumor deposits**						
Without	1			1		
With	< 0.01*	5.60	2.98–10.55	< 0.01*	7.51	3.77–14.94
**CEA level**						
Normal	1			1		
Elevated	< 0.01*	2.22	1.33–3.70	< 0.01*	3.01	1.65–5.49
**CA19-9 level**						
Normal	1			1		
Elevated	0.01*	2.15	1.18–3.92	< 0.01*	3.17	1.68–5.98
**Combined T stages**						
T_1_ + T_2_	1			1		
T_3_ + T_4_	< 0.01*	3.22	1.46–7.09	0.01*	4.08	1.46–11.40
**Combined N stages**						
N_0_	1			1		
N_1 +_ N_2_	< 0.01*	2.60	1.55–4.36	< 0.01*	2.50	1.37–4.56
**TNM stages**						
I	1			1		
II	0.09	2.35	0.88–6.23	0.04*	4.73	1.09–20.49
III	< 0.01*	4.56	1.78–11.68	0.01*	7.97	1.89–33.65
**BMI (kg/m**^**2**^**)**	0.01*	0.90	0.84–0.98	0.01*	0.89	0.82–0.98
**LFR**						
Low	1			1		
High	< 0.01*	0.27	0.14–0.51	< 0.01*	0.17	0.07–0.41

*With significant statistical difference

**Table 3:** Risk factors for DFS and OS by multivariate analysis

	DFS			OS		
	***P***	HR	95% ***CI***	***P***	HR	95% ***CI***
**Gender**						
Male	1			1		
Female	0.02*	0.48	0.26–0.86	0.03*	0.45	0.22–0.92
**Tumor deposits**						
Without	1			1		
With	< 0.01*	3.00	1.49–6.02	< 0.01*	5.36	2.62–10.98
**CEA level**						
Normal	1					
Elevated	0.05*	1.70	1.00–2.89			
**CA19-9 level**						
Normal				1		
Elevated				< 0.01*	2.54	1.33–4.85
**BMI (kg/m**^**2**^**)**				0.05*	0.92	0.84–1.00
**Combined N stages**						
N_0_	1					
N_1 +_ N_2_	0.02*	2.01	1.15–3.51			
**LFR**						
Low	1			1		
High	< 0.01*	0.32	0.16–0.61	< 0.01*	0.23	0.09–0.55

*With significant statistical difference

## Discussion

In this study, we found that the LFR could be used as a reliable prognostic indicator in nonmetastatic CRC, and its prognostic efficacy is likely to be superior to individual LC or FIB with regard to OS. Patients with a relatively low LFR had worse survival than those with a high LFR, and the LFR was an independent risk factor for the outcome in these patients. Additionally, the role of LFR in prognosis was maintained in CEA normal cases and could be effectively distinguished from those that have a poor outcome. To the best of our knowledge, this is the first report concerning the role of LFR in CRC.

It is notable that the prognostic value of LC and FIB has been validated in CRC previously but with individual limitations. For LC, Liang et al. collected 1332 stage 2 patients which included 459 patients who presented high risk of AC, and their results showed that pretreatment LC (cutoff 1300/mm^3^) was independently associated with survival [24]. In line with this, Noh et al. performed a study with 231 stages 2–3 patients who received curative surgery in addition to the subsequent FOLFOX regimen AC and suggested that LC was also independently correlated with the outcome [18]. However, the use of LC in predicting survival may limited by its relatively small AUC and inconsistent cutoff points. For example, Iseki et al. reported that the AUC for a single LC (cutoff 1700/mm^3^) in predicting DFS was 0.55, which was not statistically significant, but it was useful in predicting OS (cutoff 1100/mm^3^, *AUC* = 0.59) [19]. Similarly, Tanio et al. found that the AUC for a single LC (cutoff 1460/mm^3^) in predicting OS was only 0.55 [43]. For FIB, Silvestris et al. conducted a study with 139 metastatic cases that received bevacizumab-based therapy and found that the AUC for FIB in forecasting DFS was 0.62 and further reduced to 0.57 in predicting OS [37]. However, similar to single LC, the cutoff points for FIB were highly inconsistent as described by a systematic review and meta-analysis [34]. In recent years, some new prognostic indicators have been established based on these markers in CRC to improve prognostic efficacy. Examples have been reported, such as NLR [21], LMR [44], the FIB and NLR ratio [45], and the FIB to prealbumin ratio [41, 42]. However, it is notable that reports regarding the role of LFR in cancer are still scarce, with only a few relevant studies but only with some relevant studies. For example, Liu et al. included 375 stages 1–4 non-small cell lung cancer patients and explored the prognostic role of the FIB-to-lymphocyte percentage ratio (FLpR), and the results indicated that patients with a high FLpR would have an increased risk of death [46]. In addition, Huang et al. indicated that a high FIB to LC ratio (FLR) correlated with peritoneal dissemination in gastric cancer [47]. Though these results are not from CRC, they could also support the idea that a low LFR (equal to a high FLpR or FLR) correlates with poor outcome. Interestingly, we also found a positive correlation of FLR with LMR and PNI but a negative correlation with NLR and PLR. As the prognostic role of these markers has been extensively validated in previous reports [21, 41, 43], we believe it could partly validate the value of LFR in our study.

Mechanistically, it is well established that lymphocytes have an extensive effect in cancer, including the inhibition of occurrence and growth [11, 48], prevention of dissemination [12], and recurrence [13]. In recent years, colorectal cancer stem cells (CCSCs) or cancer-initiating cells have been identified and are thought to be the ultimate source of cancer initiation, progression, resurrection, and treatment resistance [49–51]. These cells in the circulatory system play a key role in cancer metastasis and recurrence [52, 53]. Interestingly, lymphocytes can efficiently recognize and eradicate these cells [54]. In addition, FIB has been found to have a broad effect on cancer development except for the aforementioned involvement of biological processes [27, 28]. Recently, it has also been reported that FIB in the TME can contribute to the invasiveness of glioblastoma tumor-initiating cells [55], and it can promote malignant biological tumor behavior by regulating epithelial-mesenchymal transition [56]. As in CRC, other researchers have found that FIB can coordinate with platelets in protecting cancer cells from natural killer cytotoxicity [57] and support tumor growth as well as local invasion and metastasis [58]. These functions could contribute to the support of CCSCs. Additionally, cancer-related inflammation is regarded as a hallmark of the disease [5], and some inflammatory factors can have a profound role in the development of the disease, particularly IL-6. As previous studies have indicated, peripheral blood IL-6 is significantly elevated in CRC patients [59, 60], which could contribute to T-lymphocyte cell-mediated immunosuppression [61]. As indicated in another study conducted in lung cancer, patients with high circulating IL-6 levels have significantly more T-regulatory cells and increased programmed cell death protein-1 expression on lymphocytes [62]. Notably, FIB was found to act not only as an inhibitor of lymphocyte adherence and cytotoxicity against cancer cells [63] but also as a source of induction of IL-6 [64]. Taking these studies into account, it is reasonable that patients with a low LFR could have impaired anticancer immunity (in particular those with abnormally elevated IL-6) and attenuated efficacy in killing CCSCs but with enhanced tumor aggressiveness and strengthened tumor protection, which could then lead to a poor prognosis. However, these ideas require further study.

Traditionally, CEA was a reliable prognostic indicator as recommended by ASCO in CRC [65]. However, its prognostic value is largely limited by its minimal sensitivity, as only 21–36% of patients are positive at diagnosis [66]. In addition, its efficacy is weaker in patients with type 2 diabetes or with a history of smoking [67, 68]. Some investigators have looked in normal patients for candidates for CEA, such as CA724 [69] and CA19-9 [70], and the Glasgow prognostic score [71]. However, these reports did not show the AUCs for the tested markers [69–71], and a large proportion of patients with normal CEA would also have normal CA724 (242/295) [69] and CA19-9 (333/385) [70]. In our study, the AUC for LFR in CEA normal cases in predicting DFS and OS was 0.67 and 0.75, respectively, meaning that patients with a low LFR also had a significantly inferior outcome. These results indicate that the LFR could also be a useful prognostic indicator in such a scenario.

There are still several limitations to the present study. First, the study is retrospective in nature with a relatively small sample size, and some biases are present. Second, peripheral lymphocytes are highly heterogeneous with distinct or even opposite functions, and some of these cells was have been found to have no impact on survival [72]. It would be more reasonable to sort a specific cluster, such as CD4+ or CD8+ cells and then examine the value of LFR. Third, both the LC and FIB are dynamic markers in the patients and could be affected by surgery and AC [73, 74]. Longitudinal measurements of LFR and further validation of its prognostic value are necessary in the future.

## Conclusion

Overall, our results indicated that the LFR could be regarded as a reliable prognostic indicator in nonmetastatic CRC, and that patients with a relatively low LFR have worse survival.

## Data Availability

The datasets generated or analyzed during the current study are available from the corresponding author (BY) on reasonable request.

## Abbreviations

LC: Lymphocyte count
FIB: Fibrinogen
CRC: Colorectal cancer
LFR: LC to FIB ratio
DFS: Disease-free survival
OS: Overall survival
AC: Adjuvant chemotherapy
TME: Tumor microenvironment
NLR: Neutrophil to lymphocyte ratio
LMR: Lymphocyte to monocyte ratio
AUCs: Area under the curves
CEA: Carcinoembryonic antigen
PLR: Platelet counts to lymphocyte ratio
PNI: Prognostic nutritional index
ROC: Receiver operating characteristic curve
CCSCs: Colorectal cancer stem cells
